# Supplementary material for: Mathematical modeling of COVID-19 transmission dynamics in Uganda: Implications of complacency and early easing of lockdown
Source: PLoS One. 2021 Feb 22;16(2):e0247456. doi: 10.1371/journal.pone.0247456 (PMC7899325; doi:10.1371/journal.pone.0247456)
Supplement: S1 File — (PDF) [file pone.0247456.s001.pdf]

1 Model equations

$$\frac{dS}{dt} = (1-(e+a))\pi - \frac{\beta bS(qI_a+I_s+gH)}{N} - \mu S + \tau R, \tag{1a}$$
$$\frac{dE}{dt} = e\pi + \frac{\beta bS(qI_a+I_s+gH)}{N} - \rho E, \tag{1b}$$
$$\frac{dI_a}{dt} = a\pi + r\rho E - \omega_a I_a, \tag{1c}$$
$$\frac{dI_s}{dt} = (1-(c+r))\rho E - \sigma_s I_s - \omega_s I_s, \tag{1d}$$
$$\frac{dH}{dt} = c\rho E + \omega_a I_a + \omega_s I_s - \sigma_h H - \alpha H, \tag{1e}$$
$$\frac{dR}{dt} = \alpha H - \mu R - \tau R, \tag{1f}$$

where $N = S + E + I_a + I_s + H + R$.

1.1 Model's basic properties

The epidemiological feasibility of system (1) can be guaranteed by proving that all its variables are non-negative at all time t, that is, the model's outputs with positive initial values will always remain positive at all time $t \geq 0$.

Theorem 1.1. *Suppose the model's initial values are* $S(0) > 0, E(0) > 0, I_a(0) > 0, I_s(0) > 0, H(0) > 0$ *and* $R(0) > 0$, *then the model's solutions,* $(S(t), E(t), I_a(t), I_s(t), H(t), R(t))$, *with positive initial values will remain positive for all time* $t \geq 0$.

Theorem 1.2. *The closed set* $\mathcal{D} = \left\{(S, E, I_a, I_s, H, R) \in \mathbb{R}^6_+; N \leq \frac{\pi}{\mu}\right\}$, *is positively invariant and attracts all positive solutions of the model.*

2 Existence of Equilibria points and their Stablility

In this section we present the equilibria points for the model (1) and also invesitigate their stability. By solving systemn(1) by using the force of infection, $\lambda = \frac{\beta b(qI_a+I_s+gH)}{N}$, and setting the model's time-derivatives to zero, leads to the derivation of the following equilibrium states.

I. The disease free equilibrium point ξ_0 obtained when the entire population is susceptible i.e. $\xi_0 = (S_0, E_0, I_{a_0}, I_{s_0}, H_0, R_0) = \left(\frac{\pi}{\mu}, 0, 0, 0, 0, 0\right)$.

II. The infected entry free equilibrium point ξ_1 obtained when all infected individuals are denied entry into the population obtained in section (2.2).

III. The positive endemic equilibrium $\xi^* = (S^*, E^*, I_a^*, I_s^*, H^*, R^*)$ obtained in subsection **??**.

2.1 The basic reproduction number and stability of disease free equilibrium

The basic reproduction number, $\mathcal{R}_0$ (the secondary infections generated by a COVID-19 asymptomatic and symptomatic infectious individual) is used to understand the disease dynamics and ascertain the effectiveness of implemented control strategies keeping the number of new cases low. We use the next-generation matrix method by van den Driessche and Watmough [1], to obtain the derivatives of the matrix expressions for the new infections and transition states. These are evaluated at the disease free equilibrium (ξ_0). Note that

$$F_{\xi_0} = \begin{pmatrix} 0 & b\beta q & b\beta & b\beta g \\ 0 & 0 & 0 & 0 \\ 0 & 0 & 0 & 0 \\ 0 & 0 & 0 & 0 \end{pmatrix}, \quad V_{\xi_0} = \begin{pmatrix} \rho & 0 & 0 & 0 \\ -r\rho & \omega_a & 0 & 0 \\ -(1-c-r)\rho & 0 & \sigma_s+\omega_s & 0 \\ -c\rho & -\omega_a & -\omega_s & \alpha+\sigma_h \end{pmatrix},$$

$$FV^{-1} = \begin{pmatrix} G & \frac{b\beta(g\omega_a+q\sigma_h+\alpha q)}{\omega_a(\alpha+\sigma_h)} & \frac{b\beta(\alpha+g\omega_s+\sigma_h)}{(\alpha+\sigma_h)(\sigma_s+\omega_s)} & \frac{b\beta g}{\alpha+\sigma_h} \\ 0 & 0 & 0 & 0 \\ 0 & 0 & 0 & 0 \\ 0 & 0 & 0 & 0 \end{pmatrix},$$

where

$$G = \frac{b\beta\left(\sigma_s\left(g\omega_a(c+r)+qr\sigma_h+\alpha qr\right)+\omega_a\left((1-c-r)\sigma_h+\alpha(1-c-r)+g\omega_s\right)+qr\omega_s\left(\alpha+\sigma_h\right)\right)}{\omega_a\left(\alpha+\sigma_h\right)\left(\sigma_s+\omega_s\right)}.$$

The spectral radius, ρ, of matrix FV^1 gives the reproduction number as,

$$\mathcal{R}_0 = \rho(FV^1) = \mathcal{R}_1 + \mathcal{R}_2 + \mathcal{R}_3.$$

Where,

$$\begin{aligned} \mathcal{R}_1 &= \frac{b\beta(1-c-r)}{\sigma_s+\omega_s} \\ \mathcal{R}_2 &= \frac{b\beta qr}{\omega_a} \\ \mathcal{R}_3 &= \frac{b\beta g\left((c+r)\sigma_s+\omega_s\right)}{\left(\alpha+\sigma_h\right)\left(\sigma_s+\omega_s\right)} \end{aligned}$$

The term $\frac{1}{(\omega_a+\sigma)}$ is the duration taken by an individual to exit the asymptomatic class, $\frac{1}{(\omega_s+\sigma)}$ is the duration taken by a symptomatic individual infecting the susceptible, and $\frac{1}{(\alpha+\sigma_h)}$ is the time it takes a hospitalized individual to recover or die from the infection.

The effective reproduction ratio, Re, defined as SARS-COV-2 community acquired infections caused by infectious individuals at any specific time, is derived as;

$$\mathcal{R}_e = \frac{b\beta}{\omega_a(\sigma_s+\omega_s)\left(\alpha+\sigma_h\right)}$$

The stability of the disease free equilbrium is obtained following Theorem 2 of [1]. Thus the following result is established

Theorem 2.1. *The COVID-19 disease free equilbrium is locally asymptotically stable when* $\mathcal{R}_0 < 1$ *and unstable otherwise.*

2.2 Entry free endemic equilibria point

This equilibria points exists when the parameters a, e are set to zero. Let $\lambda * = \frac{\beta b(qI_a^* + I_s^* + H^*)}{N^*}$ indicated the force of infection at steady state. Then the following polynomial is obtained by solving system (1) and substititing the solution into λ^*. Thus,

$$A_3\lambda^{*2} + A_2\lambda^* + A_1 = 0, \tag{2}$$

where

$$\begin{aligned}
A_1 &= -\frac{\pi b\beta\mu(\mu+\tau)\left(\sigma_s\left(g\omega_a(a+e(c+r))+k_4q\right)+\omega_s\left(g(a+e)\omega_a+k_4q\right)+ek_2\omega_a\left(\alpha+\sigma_h\right)\right)}{\omega_a}, \\
A_2 &= \frac{\pi\lambda\left(\sigma_s\left((\mu+\tau)\left(G_1\omega_a-G_5\rho\sigma_h\right)-\alpha G_4\rho\right)+\omega_a\left(G_2(\mu+\tau)+\alpha k_2k_6\rho\right)+\rho\omega_s\right)}{\rho\omega_a}, \\
A_3 &= \frac{\pi\lambda^2\left(\sigma_s\left(C_1\omega_a+\alpha\rho\left(a\left(k_2\tau+\mu-\mu r\right)\right)+r(\mu+\tau)\right)+\sigma_h(\mu+\tau)\right)+C_3\omega_a+C_2\rho\omega_s)}{\rho\omega_a},
\end{aligned}$$

where

$$\begin{aligned}
k_4 &= (a+er)(\alpha+\sigma_h), \quad k_5 = a\rho(1-r)+(1-a)\omega_a+\rho r, \quad k_6 = -b\beta((1-a)\mu+\tau)+e\mu(\mu+\tau) \\
C_1 &= \alpha\left(\rho\left(ak_2+c+r\right)-a\mu+\mu+\tau\right)+\rho(\mu+\tau)\left(ak_2+c+r\right) \\
C_2 &= (a(1-r)+r)\sigma_h(\mu+\tau)+\alpha(a(\mu-\mu r)+r(\mu+\tau)) \\
C_3 &= (1-a)\sigma_h(\mu+\tau)\left(k_2\rho+\omega_s\right)+\alpha k_2\rho((1-a)\mu+\tau)+\omega_s(\alpha(-a\mu+\mu+\rho+\tau)+\rho(\mu+\tau)) \\
G_1 &= \rho\left(-(1-a)b\beta g(c+r)-ab\beta g+\mu(a+e(c+r))+\alpha-\alpha ek_2\right)+\alpha e\mu+\sigma_h\left(e\mu+k_1\rho\right) \\
G_2 &= \omega_s\left(\rho(\mu(a+e)+\alpha-b\beta g)+\alpha e\mu+\sigma_h\left(e\mu+k_1\rho\right)\right)-k_2\rho\sigma_h((1-a)b\beta-e\mu) \\
G_3 &= -\sigma_h(\mu+\tau)(b\beta q(a(1-r)+r)-\mu(a+er))-a\alpha\mu(b\beta q(1-r)-\mu-\tau)-\alpha r(\mu+\tau)(b\beta q-e\mu) \\
G_4 &= ab\beta k_2q\tau+a\mu(b\beta q(1-r)-\mu-\tau)+r(\mu+\tau)(b\beta q-e\mu) \\
G_5 &= b\beta q(a(-r)+a+r)-\mu(a+er)
\end{aligned}$$

When $a = 0,\ e = 0,\ \lambda^* \neq 0$, a unique entry free equilbrium point ξ_1 is obtained. In this case, $A_1 = 0$ and the force of infection polynomial reduces to $\lambda^*(A_3\lambda^* + A_2) = 0$ where

$$A_2 = \pi(\mu+\tau)\left(\alpha+\sigma_h\right)\left(\sigma_s+\omega_s\right)\left(1-\mathcal{R}_0\right).$$

Which is negative if $\mathcal{R}_0 > 1$. With $A_3 > 0$, then either $\lambda^* = 0$ or $\lambda^* = -\dfrac{A_2}{A_3}$. Thus, the posive entry free endemic equilbriun will be given as;

$$
\begin{aligned}
S_1 &= \frac{\pi(\mu+\tau)\,(\alpha+\sigma_h)\,(\sigma_s+\omega_s)}{Q_1\sigma_s+Q_2\omega_s},\\
E_1 &= \frac{\pi\lambda^*(\mu+\tau)\,(\alpha+\sigma_h)\,(\sigma_s+\omega_s)}{\rho\,(Q_1\sigma_s+Q_2\omega_s)},\\
I_{a_1} &= \frac{\pi\lambda^* r(\mu+\tau)\,(\alpha+\sigma_h)\,(\sigma_s+\omega_s)}{\omega_a\,(Q_1\sigma_s+Q_2\omega_s)},\\
I_{s_1} &= \frac{\pi k_2\lambda^*(\mu+\tau)\,(\alpha+\sigma_h)}{Q_1\sigma_s+Q_2\omega_s},\\
H_1 &= \frac{\pi\lambda^*(\mu+\tau)\,((c+r)\sigma_s+\omega_s)}{Q_1\sigma_s+Q_2\omega_s},\\
R_1 &= \frac{\pi\alpha\lambda^*\,((c+r)\sigma_s+\omega_s)}{Q_1\sigma_s+Q_2\omega_s},
\end{aligned}
$$

where

$$
\begin{aligned}
k_1 &= 1-a-e; \quad k_2 = 1-c-r; \quad k_3 = a\mu + a\lambda^*(1-r) + r(e\mu+\lambda^*);\\
Q_1 &= \alpha\mu(\mu+\tau) + \alpha\lambda^*(\tau k_2+\mu) + \sigma_h(\lambda^*+\mu)(\mu+\tau)\\
Q_2 &= \alpha\mu(\lambda^*+\mu+\tau) + \sigma_h(\lambda^*+\mu)(\mu+\tau)
\end{aligned}
$$

2.3 Local stability of the entry free endemic equilibrium ξ_1

To determine the local stability of the entry free equilibrium ξ_1, we use the center manifold theorem 4.1 described in [4]. To apply the center manifold thoerem, we consider a change of variable such that $S = x_1, E = X_2, I_a = x_3, I_s = x_4, H = x_5, R = X_6$ such that system 1 is transformed as;

$$
\begin{aligned}
\frac{dS}{dt} &= (1-(e+a))\pi - \frac{\beta b x_1(qx_3+x_4+gx_5)}{N} - \mu x_1 + \tau x_6, && \text{(3a)}\\
\frac{dE}{dt} &= e\pi + \frac{\beta b x_1(qx_3+x_4+gx_5)}{N} - \rho x_2, && \text{(3b)}\\
\frac{dI_a}{dt} &= a\pi + r\rho x_2 - \omega_a x_3, && \text{(3c)}\\
\frac{dI_s}{dt} &= (1-(c+r))\rho x_2 - \sigma_s x_4 - \omega_s x_4, && \text{(3d)}\\
\frac{dH}{dt} &= c\rho x_2 + \omega_a x_3 + \omega_s x_4 - \sigma_h x_5 - \alpha x_5, && \text{(3e)}\\
\frac{dR}{dt} &= \alpha x_5 - \mu x_6 - \tau x_6, && \text{(3f)}
\end{aligned}
$$

where

$$N = x_1 + x_2 + x_3 + x_4 + x_5 + x_6$$

We compute the Jacobian matrix evaluated at disease free equilibrium as

$$J_{\xi_0} = \begin{pmatrix} -\mu & 0 & -b\beta q & -b\beta & -b\beta g & \tau \\ 0 & -\rho & b\beta q & b\beta & b\beta g & 0 \\ 0 & \rho r & -\omega_a & 0 & 0 & 0 \\ 0 & (1-c-r)\rho & 0 & -\sigma_s - \omega_s & 0 & 0 \\ 0 & c\rho & \omega_a & \omega_s & -\alpha - \sigma_h & 0 \\ 0 & 0 & 0 & 0 & \alpha & -\mu - \tau \end{pmatrix}$$

Note that the matrix J_{ξ_0} has a simple zero eigenvalue if β is taken as the bifurcation parameter obtained by setting $\mathcal{R}_0 = 1$ such that

$$\beta^* = \frac{\omega_a (\alpha + \sigma_h)(\sigma_s + \omega_s)}{b\left(\sigma_s \left(g\omega_a(c+r) + qr(\alpha + \sigma_h)\right) + \omega_a \left(g\omega_s + k_2(\alpha + \sigma_h)\right) + qr\omega_s(\alpha + \sigma_h)\right)}$$

Thus, following Theorem 4.1 in [4], it can be shown that J_{ξ_0} has the left eigenvector associated with the zero eigenvalue as $v = (v_1, v_2, v_3, v_4, v_5, v_5)^T$ where;

$$\begin{aligned} v_1 &= 0, \qquad v_2 > 0, \qquad v_3 = b\beta v_2 \left(\frac{g}{\alpha + \sigma_h} + \frac{q}{\omega_0} \right), \\ v_4 &= \frac{b\beta v_2 (\alpha + g\omega_s + \sigma_h)}{(\alpha + \sigma_h)(\sigma_s + \omega_s)}, \qquad v_5 = \frac{b\beta g v_2}{\alpha + \sigma_h}, \qquad v_6 = 0. \end{aligned}$$

and the right eigenvector associated with the zero eigenvalue as $w = (w_1, w_2, w_3, w_4, w_5, w_6)^T$ where;

$$\begin{aligned} w_1 &= \frac{w_6 \left(-\frac{b\beta g(\mu+\tau)}{\alpha} - \frac{b\beta qr(\mu+\tau)(\alpha+\sigma_h)(\sigma_s+\omega_s)}{\alpha\omega_0(r\sigma_s+\omega_s)} + \frac{b\beta(r-1)(\mu+\tau)(\alpha+\sigma_h)}{\alpha(r\sigma_s+\omega_s)} + \tau \right)}{\mu}, \\ w_2 &= \frac{w_6(\mu+\tau)(\alpha+\sigma_h)(\sigma_s+\omega_s)}{\alpha\rho(r\sigma_s+\omega_s)}, \\ w_3 &= \frac{rw_6(\mu+\tau)(\alpha+\sigma_h)(\sigma_s+\omega_s)}{\alpha\omega_a(r\sigma_s+\omega_s)}, \qquad w_4 = \frac{(1-r)w_6(\mu+\tau)(\alpha+\sigma_h)}{\alpha(r\sigma_s+\omega_s)}, \\ w_5 &= \frac{w_6(\mu+\tau)}{\alpha}, \qquad w_6 > 0, \end{aligned}$$

The non zero partial derivatives for computing coefficients **a** and **b** are

$$\frac{\partial f_1}{\partial\beta^*\partial x_3} = -bq, \quad \frac{\partial f_1}{\partial\beta^*\partial x_4} = -b, \quad \frac{\partial f_1}{\partial\beta^*\partial x_5} = -bg, \quad \frac{\partial f_2}{\partial\beta^*\partial x_3} = bq, \frac{\partial f_2}{\partial\beta^*\partial x_4} = b, \frac{\partial f_2}{\partial\beta^*\partial x_5} = bg.$$

$$
\begin{aligned}
\frac{\partial f_2}{\partial x_2 \partial x_3} &= -\frac{b\beta\mu q}{\pi}, \quad \frac{\partial f_2}{\partial x_2 \partial x_4} = -\frac{b\beta\mu}{\pi}, \quad \frac{\partial f_2}{\partial x_2 \partial x_5} = -\frac{b\beta g\mu}{\pi}, \quad \frac{\partial f_2}{\partial x_3 \partial x_2} = -\frac{b\beta\mu q}{\pi}, \\
\frac{\partial f_2}{\partial x_3 \partial x_3} &= -\frac{2b\beta\mu q}{\pi}, \frac{\partial f_2}{\partial x_3 \partial x_4} = -\frac{b\beta\mu(q+1)}{\pi}, \quad \frac{\partial f_2}{\partial x_3 \partial x_5} = -\frac{b\beta\mu(g+q)}{\pi}, \quad \frac{\partial f_2}{\partial x_3 \partial x_6} = -\frac{b\beta\mu q}{\pi} \\
\frac{\partial f_2}{\partial x_4 \partial x_2} &= -\frac{b\beta\mu}{\pi} \quad \frac{\partial f_2}{\partial x_4 \partial x_3} = -\frac{b\beta\mu(q+1)}{\pi}, \quad \frac{\partial f_2}{\partial x_4 \partial x_4} = -\frac{2b\beta\mu}{\pi}, \quad \frac{\partial f_2}{\partial x_4 \partial x_5} = -\frac{b\beta(g+1)\mu}{\pi}, \\
\frac{\partial f_2}{\partial x_4 \partial x_6} &= -\frac{b\beta\mu}{\pi}, \quad \frac{\partial f_2}{\partial x_5 \partial x_2} = -\frac{b\beta g\mu}{\pi} \quad \frac{\partial f_2}{\partial x_5 \partial x_3} = -\frac{b\beta\mu(g+q)}{\pi}, \quad \frac{\partial f_2}{\partial x_5 \partial x_4} = -\frac{b\beta(g+1)\mu}{\pi} \\
\frac{\partial f_2}{\partial x_5 \partial x_5} &= -\frac{2b\beta g\mu}{\pi}, \quad \frac{\partial f_2}{\partial x_5 \partial x_6} = -\frac{b\beta g\mu}{\pi}, \quad \frac{\partial f_2}{\partial x_6 \partial x_3} = -\frac{b\beta\mu q}{\pi}, \quad \frac{\partial f_2}{\partial x_6 \partial x_4} = -\frac{b\beta\mu}{\pi}, \\
\frac{\partial f_2}{\partial x_6 \partial x_5} &= -\frac{b\beta g\mu}{\pi}.
\end{aligned}
$$

Thus,

$$
\begin{aligned}
\mathbf{a} &= -\frac{2b\beta\mu v_2 (w_2 + w_3 + w_4 + w_5 + w_6)(g w_5 + q w_3 + w_4)}{\pi} < 0. \\
\mathbf{b} &= b v_2 (g w_5 + q w_3 + w_4) > 0.
\end{aligned}
$$

Since $v_2 > 0$ and $w_2, w_3, w_4, w_5, w_6 > 0$, then by Theorem 4.1 (iv) in [4], the entry free endemic equilibrium is locally asymptotically stable. In this case, if SARS-CoV2 infected individuals are denied entry into a community, then the disease can only invade at a very low endemic level if $\mathcal{R}_0 > 1$. If $\mathcal{R}_0$ drops below unity again, the disease disappears from the population.

2.4 Endemic equilbrium Point ξ^*

When $a \neq 0,\ e \neq 0,\ \lambda^* \neq 0$, then the polynomial (2) is solved to obtain the equilbrium point ξ^*. Note that $A_3 > 0$ and $A_1 < 0$ indicating that irrespective of the sign of A_2, there will always be one positive root of (2). substit
iting λ^* into the solution of (1) set to zero, we obtain ξ^* as;

$$
\begin{aligned}
S^* &= \frac{\pi\left(\sigma_s\left(\alpha\tau\left(ek_2+1\right)+k_1\sigma_h(\mu+\tau)+\alpha k_1\mu\right)+\omega_s\left(\alpha\tau+k_1\sigma_h(\mu+\tau)+\alpha k_1\mu\right)\right)}{Q_1\sigma_s+Q_2\omega_s}, \\
E^* &= \frac{\pi\left(\sigma_s+\omega_s\right)\left(\alpha(\lambda(-a\mu+\mu+\tau)+e\mu(\mu+\tau))+\sigma_h(\mu+\tau)((1-a)\lambda+e\mu)\right)}{\rho\left(Q_1\sigma_s+Q_2\omega_s\right)}, \\
I_a^* &= \frac{\pi\left(\sigma_s\left(\alpha\left(a\lambda\left(k_2\tau+\mu(1-r)\right)+a\mu(\mu+\tau)+r(\mu+\tau)(e\mu+\lambda)\right)+k_3\sigma_h(\mu+\tau)\right)+Q_3\omega_s\right)}{\omega_a\left(Q_1\sigma_s+Q_2\omega_s\right)}, \\
I_s^* &= \frac{\pi k_2\left(\alpha(\lambda((1-a)\mu+\tau)+e\mu(\mu+\tau))+\sigma_h(\mu+\tau)((1-a)\lambda+e\mu)\right)}{Q_1\sigma_s+Q_2\omega_s}, \\
H^* &= \frac{\pi(\mu+\tau)\left(\sigma_s\left(a\left(\lambda k_2+\mu\right)+(c+r)(e\mu+\lambda)\right)+\omega_s(\mu(a+e)+\lambda)\right)}{Q_1\sigma_s+Q_2\omega_s}, \\
R^* &= \frac{\pi\alpha\left(\sigma_s\left(a\lambda k_2+a\mu+(c+r)(e\mu+\lambda)\right)+\omega_s(\mu(a+e)+\lambda)\right)}{Q_1\sigma_s+Q_2\omega_s},
\end{aligned}
$$

where

$$
Q_3 = \sigma_h(\mu+\tau)(a\mu+a\lambda^*(1-r)+r(e\mu+\lambda^*))+\alpha(a\mu(\mu+\lambda^*(1-r)+\tau)+r(\mu+\tau)(e\mu+\lambda^*)).
$$

References

[1] P. van den Driessche and J. Watmough (2002). Reproduction numbers and sub-threshold endemic equilibria for compartmental models of disease transmission. *Math. Biosci.*, 180:29–48.

[2] Lakshmikantham, S., Leela, S., and Martynyuk, A.A. (1989). Stability analysis of nonlinear systems. *New York; Basel: Marcel Dekker, Inc.*

[3] LaSalle, J.P., (1976). The stability of dynamical systems. *Society for Industrial and Applied Mathematics, Philadelphia.*

[4] Castillo-Chavez C. and Song B., (2004). Dynamical models of Tuberculosis and their application. *Math. Biosci. Eng.*, 1:361–404.

[5] Strogatz, S. (2001). Nonlinear dynamics and chaos: with applications to physics, biology, chemistry, and engineering. *Boulder: Westview Press.*
