# Supplementary material for: Mathematical modeling of COVID-19 transmission dynamics in Uganda: Implications of complacency and early easing of lockdown
Source: PLoS One. 2021 Feb 22;16(2):e0247456. doi: 10.1371/journal.pone.0247456 (PMC7899325; doi:10.1371/journal.pone.0247456)
Supplement: Attachment — Submitted filename: Report PLoS ONE.docx [file pone.0247456.s002.docx]

Report on the “Mathematical modelling of COVID-19 transmission dynamics in Uganda: implications of complacency and early easing of lockdown” by Joseph Y.T. Mugisha, Joseph Ssebuliba, Juliet N. Nakakawa, Cliff R. Kikawa, and Amos Ssematimba

1. A standard presentation of this kind of model usually includes derivation of R­_0_, the basic reproduction number, equilibrium points, and stability analysis and these should be done mathematically (not through simulation or numerically). Since the focus of this paper is the dynamics that are mainly observed through simulations, the authors can present the mathematical aspect in a very brief way. But this is very important for this kind of modeling.
2. Possibility and impact of a second wave is not clear. I do not understand how this second wave is accommodated in the model. If the authors intend to provide a time line when this second wave might hit, that would be great. However, the objective and presentation can be made much more explicit. This possibility of the second wave is an important issue in this paper. So clear understanding and prediction of time and extent, if possible, should be presented and justified properly.
3. There is no justification of the sample size N=300 in sensitivity analysis.
4. Few minor English corrections are required, like at page 95: Write “since” instead of “from”, etc.
