# Supplementary material for: Mathematical modeling of COVID-19 transmission dynamics in Uganda: Implications of complacency and early easing of lockdown
Source: PLoS One. 2021 Feb 22;16(2):e0247456. doi: 10.1371/journal.pone.0247456 (PMC7899325; doi:10.1371/journal.pone.0247456)
Supplement: Attachment — Submitted filename: Mugisha et al_rebuttal.docx [file pone.0247456.s003.docx]

Department of Mathematics

Faculty of Science

Gulu University

P.O. Box 166

Gulu, Uganda.

October 22, 2020

Dr. Siew Ann Cheong
Academic Editor,

PLOS ONE

Dear Dr. Cheong,

Re: **Revision of manuscript number PONE-D-20-24477 titled “Mathematical modeling of COVID-19 transmission dynamics in Uganda: Implications of Complacency and Early Easing of Lockdown”**

We wish to submit a revised version of the above mentioned manuscript in which we have addressed the comments raised during the review process, both by the reviewers and the editor.

We appreciate your and the reviewers’ efforts towards improving this manuscript and also appreciate the opportunity to have our work published in PLOS ONE journal.

In the text below, we include our responses in red bold fonts below each point raised.

Thank you.

Sincerely,

Amos Ssematimba, Ph.D

(On behalf of the co-authors)

=====================================================================

**Comments to the Author**

**Reviewer #1:**

Abstract should be structural manner including of subsection like background, methods, results, discussion.

**This observation is appreciated although only slight amendments have been made. Generally, the abstract was drafted in such a way that the different paragraphs capture content for the different subsections albeit without specific headings. There is one paragraph each for Background, Materials and Methods, Results and Discussion in accordance with PLOS ONE journal guidelines to authors.**


The introduction section produces redundant information on COVID-19 background which could be no interest for readers. Rather it is recommended to present related modelling works. Some of works the suggestive
a) COVID-19 disease outbreak forecasting of registered and recovered cases after sixty-day lockdown in Italy: A data driven model approach
b) A Simulation of a COVID-19 Epidemic Based on a Deterministic SEIR Model
c) Modified SEIR and AI prediction of the epidemics trend of COVID-19 in China under public health interventions

**The introduction section has been improved upon and paragraphs dedicated to COVID-19 modelling have been introduced as suggested by the reviewer and the proposed articles have been utilized. These edits are captured on page 4 lines 68-77 as “…The role of compartmental mathematical models in predicting COVID-19 dynamics under varying interventions has been demonstrated. Examples of such studies include, Yang et al. [19] who effectively predicted the epidemic peaks and sizes in China using an approach that integrated population migration and COVID-19 epidemiological data into a Susceptible-Exposed-Infectious-Removed (SEIR) model together with an artificial intelligence approach. Also, Carcione et al. [21] demonstrated how mathematical models can be used to generate meaningful insights amidst epidemiological and etiological data challenges. They simulated a COVID-19 epidemic for Italy based on an SEIR model and they emphasized the importance of assessing the effectiveness of lockdowns on epidemic burden. …”**


Clarity of figures is missing. Please enhance the figure aspect ratios

**The quality of the figures has been improved upon.**

**Reviewer #2:**

1. A standard presentation of this kind of model usually includes derivation of R¬0, the basic reproduction number, equilibrium points, and stability analysis and these should be done mathematically (not through simulation or numerically). Since the focus of this paper is the dynamics that are mainly observed through simulations, the authors can present the mathematical aspect in a very brief way. But this is very important for this kind of modeling.

**The qualitative analysis of the model was done earlier but the write of the paper was hinged more on the quantitative analysis give the original objective. However, on the reviewer’s recommendation, we have now reintroduced the content on the qualitative analysis both in the main text and as supplementary information. The detailed analysis steps and all other results have been included as supporting information in file S1 for completeness.**

**We now have a brief description of methods in Section 3.1 page 9 lines 167-174 as “…Qualitative analysis of the model: The model was analyzed qualitatively to generate insightful thresholds that can inform public health policy decisions. Under this analysis, the biological feasibility of the model was assessed, the expression for the basic reproduction number (R_0_) defined as the secondary infections generated by a typical COVID-19 infectious individual when introduced in a naïve population was derived using the next generation matrix method [27] and equilibrium points were determined and their stability analyzed. Summarized key results of these analyses are presented under the results’ section and the detailed approaches and all other findings are included in the supporting information file S1….”**

**A summary of key results on this analysis has been introduced in section 4.1 page 11 in lines 224-236 as “…On qualitative analysis of the model: The basic reproduction number is given by** $\boldsymbol{R}_{\boldsymbol{0}}\boldsymbol{=}\boldsymbol{R}_{\boldsymbol{1}}\boldsymbol{+}\boldsymbol{R}_{\boldsymbol{2}}\boldsymbol{+}\boldsymbol{R}_{\boldsymbol{3}}\boldsymbol{,}$ **where** $\boldsymbol{R}_{\boldsymbol{1}}\boldsymbol{=}\frac{\boldsymbol{b \beta(1-c-r)}}{\boldsymbol{\sigma}_{\boldsymbol{s}}\boldsymbol{+}\boldsymbol{\omega}_{\boldsymbol{s}}}$ **is the reproductive rate of symptomatic individuals,** $\boldsymbol{R}_{\boldsymbol{2}}\boldsymbol{=}\frac{\boldsymbol{b \beta r q}}{\boldsymbol{\omega}_{\boldsymbol{a}}}$ **is the reproductive rate of asymptomatic individuals and** $\boldsymbol{R}_{\boldsymbol{3}}\boldsymbol{=}\frac{\boldsymbol{b g \beta(}\left( \boldsymbol{c+r} \right)\boldsymbol{\sigma}_{\boldsymbol{s}}\boldsymbol{+}\boldsymbol{\omega}_{\boldsymbol{s}}\boldsymbol{)}}{\left( \boldsymbol{\alpha+}\boldsymbol{\sigma}_{\boldsymbol{h}} \right)\boldsymbol{(}\boldsymbol{\sigma}_{\boldsymbol{s}}\boldsymbol{+}\boldsymbol{\omega}_{\boldsymbol{s}}\boldsymbol{)}}$ **is the reproductive rate of hospitalized individuals. Additionally,** $\frac{\boldsymbol{1}}{\boldsymbol{\omega}_{\boldsymbol{a}}}$ **is the time that an individual spends in the asymptomatic class,** $\frac{\boldsymbol{1}}{\boldsymbol{\sigma}_{\boldsymbol{s}}\boldsymbol{+}\boldsymbol{\omega}_{\boldsymbol{s}}}$ **is the time that an individual spends in the symptomatic class and** $\frac{\boldsymbol{1}}{\left( \boldsymbol{\alpha+}\boldsymbol{\sigma}_{\boldsymbol{h}} \right)}$ **is the time a hospitalized individual spends in hospital.**

**The biological feasibility of model system was guaranteed by proving that all its variables are non-negative at all time t, i.e., the model's outputs with positive initial values will always remain positive at all time t > 0. On the existence and stability of equilibrium points, the disease free equilibrium point was found to be locally asymptotically stable when R_0_ < 1 and unstable otherwise. The analysis further revealed that if all infected travelers were denied entry into the country, then the disease could only invade at a very low endemic level if R_0_ > 1. For further analytical details on these, refer to the supporting information file S1….”**

2. Possibility and impact of a second wave is not clear. I do not understand how this second wave is accommodated in the model. If the authors intend to provide a time line when this second wave might hit, that would be great. However, the objective and presentation can be made much more explicit. This possibility of the second wave is an important issue in this paper. So clear understanding and prediction of time and extent, if possible, should be presented and justified properly.

**There was lack of clarity in presenting aspects about the possibility and impact of a second wave and we have now improved upon the flow of content on this aspect and the manuscript in general.**

**On the origin and cause of the 'second wave', note that this study in part aimed to evaluate the impact of the intervention measures that were implemented by the Government of Uganda that included lockdown of almost all nonessential activities in the whole country. This study aimed to generate insight into how best the instituted lockdown could be lifted (i.e., when and what percentage to be optimally lifted from lockdown) while averting the second wave. Technically, the predicted second wave is an exogenous event resulting specifically from a hurried partial easing of lockdown when there still are untraced infectious individuals freely living in the community no matter how few. Embedded in the parameter b (i.e. percentage of susceptible individuals that is available) that captures the proportion of people that is out of lockdown and is therefore “mingling” or interacting freely.**

**We have added the following in the methods section on page 10 lines 199-202 as “…This simulation was intended to determine when it would be “safe” to allow up to 75% of the population to resume their normal activities without having to worry about the possibility of having a second wave of infection that can be initiated by the few remnant infectious individuals in the community…”**

**In the Discussion section we mention the that the second wave was a consequence of easing lockdown on page 17 lines339-342 as “The results in Figure 5 reveal that, even in a fairly ideal situation with no new arrivals of imported cases nor the occurrence of hospital acquired infections, once the lockdown is hurriedly lifted to a 75% level, the yet-to-be detected cases in the community have potential to start a second and more disastrous epidemic wave.…”**

3. There is no justification of the sample size N=300 in sensitivity analysis.

**Note that N denotes the sample size and the key requirement it that its value be greater than the number of parameters varied plus 1 (i.e. j+1) but recommended to be set to a large enough value to guarantee precision while bearing in mind the need for maintaining computer run time and efficiency. N=300 gave us both precision and efficiency. We have added a justification in the main text page 11 lines 218-219 as “…a sample size of** $\boldsymbol{N=300}$ **(set to this value to guarantee precision of the outcome)…”**

4. Few minor English corrections are required, like at page 95: Write “since” instead of “from”, etc.

**This and other textual edits have been made.**

6. PLOS authors have the option to publish the peer review history of their article ([what does this mean?](https://journals.plos.org/plosone/s/editorial-and-peer-review-process#loc-peer-review-history)). If published, this will include your full peer review and any attached files.


If you choose “no”, your identity will remain anonymous but your review may still be made public.


**Do you want your identity to be public for this peer review?** For information about this choice, including consent withdrawal, please see our [Privacy Policy](https://www.plos.org/privacy-policy).

Reviewer #1: No

Reviewer #2: **Yes:** Indranil Mukhopadhyay

[NOTE: If reviewer comments were submitted as an attachment file, they will be attached to this email and accessible via the submission site. Please log into your account, locate the manuscript record, and check for the action link "View Attachments". If this link does not appear, there are no attachment files.]

While revising your submission, please upload your figure files to the Preflight Analysis and Conversion Engine (PACE) digital diagnostic tool, <https://pacev2.apexcovantage.com/>. PACE helps ensure that figures meet PLOS requirements. To use PACE, you must first register as a user. Registration is free. Then, login and navigate to the UPLOAD tab, where you will find detailed instructions on how to use the tool. If you encounter any issues or have any questions when using PACE, please email PLOS at [figures@plos.org](mailto:figures@plos.org). Please note that Supporting Information files do not need this step.
