# Supplementary material for: Mathematical modeling of COVID-19 transmission dynamics in Uganda: Implications of complacency and early easing of lockdown
Source: PLoS One. 2021 Feb 22;16(2):e0247456. doi: 10.1371/journal.pone.0247456 (PMC7899325; doi:10.1371/journal.pone.0247456)
Supplement: Attachment — Submitted filename: Rebuttal_Mugisha et al 2020.docx [file pone.0247456.s004.docx]

**15 December 2020**

**Revision of manuscript number PONE-D-20-24477R1 titled “Mathematical modeling of COVID-19 transmission dynamics in Uganda: Implications of Complacency and Early Easing of Lockdown”**

**On behalf of the co-authors, we appreciate all your efforts devoted towards improving this manuscript. Thank you so much!**

**Review Comments to the Author**

Please use the space provided to explain your answers to the questions above. You may also include additional comments for the author, including concerns about dual publication, research ethics, or publication ethics. (Please upload your review as an attachment if it exceeds 20,000 characters)

**Reviewer #1**: I wondering that authors did not address the specific comments rasied by reviewer that might considered as not a good practice.
Abstract section should has to mention the sub section and we can not assume that readers will automatically understand the authors view.

**As requested, we have introduced section headers in the abstract on page 1 in lines 14-21.**

Authors has to address the established data modelling approches in non ease of lockdown and compare it with some spcial case studies like Italy and other european countries (COVID-19 disease outbreak forecasting of registered and recovered cases after sixty-day lockdown in Italy: A data driven model approach).

**We have addressed this by introducing content comparing pre- and post- intervention measures in the introduction as on page 4 in lines 80-84 as “… Relatedly, Chintalapudi et al.[21] concluded basing on outcomes from their data driven model analysis that if the Italian government had extended the March 2020 country lockdown and self-isolation measures by 60 days, the number of registered cases would decrease by 35% while those recovered would increase by 66%. All these approaches demonstrate the utility of mathematical models in assessing the impact of intervention measures on COVID-19 dynamics. …” Relatedly, our study assesses the impact of lockdown through assessing the disease burden (number of new cases and number hospitalized) under different lockdown percentages (10%, 30% and 50%) as depict in Figure 2.**

**Following the reviewer advice, we also deemed it prudent to add more related literature. We added content from another relevant study by Rawson et al. (2020) on page 4 in lines 84-87 as “… On using modeling to assess how efficiently lockdowns can be eased in the United Kingdom, Rawson et al. [22] applied an optimal control framework to an adapted SEIR model framework and found that the optimal strategy was a phased-out release strategy. …”**

**We also added related content in the discussion section on page 17 in lines 352-355 as “… On the importance of a phased-out release of lockdown, a modeling study parametrized for the United Kingdom reported that the optimum strategy was to release approximately half of the population 2 to 4 weeks from the end of an initial infection peak and then wait another 3 to 4 months to release everyone [Rawson et al. 2020]. …”**

Some areas spell check and grammetical errors has to address

**These have been addressed.**

Best wishes

**Reviewer #3**: The manuscript utilises a compartmental SEIHR framework to assess the disease dynamics in Uganda, and consider how to tackle future infection possibilities. Current manuscript revisions have already improved the manuscript well. While I feel the work has great potential to advance the understanding of disease dynamics within Uganda, I have substantial concerns with the current model formulation, and the conclusions the authors draw.

The primary issue is that the authors attempt to, arguably, do too much with the model, and in doing so each conclusion suffers. I list below my general concerns with the model formulation, and which scenarios are ill-posed for the current model. In general I would urge the authors to restrict their scope, and tweak the model to draw stronger conclusions on a specific research question or two. I feel the model may be best suited with minor adjustment to assessing “The possibility of a second wave infection”, or “The impact of lockdown measures”, while it is currently not an appropriate model for assessing “The impact of magnitude of imported cases” or “Impact of hospital acquired infections”.
The Introduction is very strong and suitably frames the work.

**We agree that trimming and refocusing the content tremendously improves that quality of the manuscript and as suggested by the reviewer, the manuscript has been refocused to address three specific questions namely; 1) assessing the potential impact of the implemented lockdown measures, 2) the dynamic impact of constant external disease pressure (phrasing suggested by the reviewer in the later comments), and 3) the possibility of the second wave of infection. We also maintain the section on sensitivity analysis.**

General Concerns
My initial concerns are with the model parameters (Table 1). Many parameters cite the Ugandan data portal (reference [4]) for their values, however I cannot locate these model parameters, nor can I infer them from Ugandan case data.

**For reliability of the study outcomes in informing Uganda policy interventions to manage the disease, it was necessary that the study uses localized parameters whenever possible. However, as was the case with other earlier modeling studies, only scanty information about the pathogen and the disease in general was available and more is just being generated as the pandemic progresses. Therefore, some of the earlier simulation studies utilized the limited data together with sensitivity analyses to infer disease dynamics under varying intervention scenarios. It is hoped that as more knowledge about the pathogen and its transmission pathways in generated, the models will be updated accordingly.**

**For our Ugandan case, not all required parameters have been documented in citable literature and this was a hurdle to the current study. For example, besides the number of new cases, recoveries and deaths that are mandatorily documented and forwarded to WHO among other stakeholders, the other parameters that cite the Ministry of Health data (reference 4) are mainly inferred or derived from the routine addresses by the personnel from the Ministry of Health, Uganda virus research institute and the office of the presidency among others and are based on observational data. Examples of such include the arrival rate of truck drivers (both infected and non-infected), number of returning residents through airports, time spent in hospital for the admitted cases which partly informs the model recovery rate, the proportion of asymptomatic infected individuals etc. In our parameter estimation, content from these routine national addresses and press releases which is often summarized in news outlets was used to infer some of the model parameters. One of such briefings can be found online at** <https://www.health.go.ug/document/update-on-covid-19-response-in-uganda/> **and** https://www.health.go.ug/covid/category/press-release/ **Studies are being conducted and it is hoped that such new studies will have data from which other more precise parameters can be obtained. For example the recently published Kirenga et al. (2020) “Characteristics and outcomes of admitted patients infected with SARSCoV-2 in Uganda”** <https://www.ncbi.nlm.nih.gov/pmc/articles/PMC7477797/pdf/bmjresp-2020-000646.pdf> **validates some of the parameter estimates.**

**We have checked the literature again and identified some articles that we could cite for some of the parameters in Table 1. For recruitment rate [Ministry of Health -Uganda. UPDATE ON THE COVID-19 OUTBREAK IN UGANDA available online at** <https://www.health.go.ug/covid/category/press-release/> **accessed on 1 December 2020. 2020.], for recovery rate of hospitalized individuals [WHO-China. Report of the WHO-China Joint Mission on Coronavirus Disease 2019, available online at** <https://www.who.int/docs/default-source/coronaviruse/who-china-joint-mission-on-covid-19-final-report.pdf>, **accessed 3 June 20202020.] and for percentage of latently infected individuals in community that becomes asymptomatic [Daniel P. Oran A, Eric J. Topol. Prevalence of Asymptomatic SARS-CoV-2 Infection. Annals of Internal Medicine. 2020;173(5):362-7. doi: 10.7326/m20-3012 %m 32491919.]**

Some parameters are also very far from similar parameter estimates in the literature. For example, a disease-induced mortality rate of 0.001 is far low than estimates elsewhere in the literature. The hospital mortality rate of 0.0001 is even more peculiar. See for example, Baud et al. (2020) “Real estimates of mortality following COVID-19 infection.”.

**The low disease-induced death rate that used in our study is for the Ugandan situation where Uganda did not register any COVID-19 related death in the first four months from the first case (i.e. March 22^nd^ to July 22^nd^ 2020). The first death was registered on 23^rd^ July 2020 and at that point, the cumulative number of cases was 1079 with 971 recoveries. In other countries, using data as of March 1^st^ 2020 (i.e. approx. 4 months (December to March) into the outbreak), Baud et al. (2020) reported mortality rate of 3·6% (95% CI 3·5–3·7) in China and 1·5% [1·2–1·7] outside of China and when adjusted for incubation period delay, mortality rates would be 5·6% (95% CI 5·4–5·8) for China and 15·2% (12·5–17·9) outside of China. Moreover, even as of December 1^st^ 2020, globally there are approximately 63.7m cases, 1.5m deaths and 44.1m recoveries while in Uganda, there have been 20,459 cases, with 205 fatalities and 8989 recoveries. These numbers may crudely indicate both the case-fatality ratio and Recovered-fatality ratio (obtained from considering only the resolved cases) are relatively lower in Uganda.**

**For completeness and clarity, we have added the following “justification” also citing Baud et al (2020) in the discussion on page 18 in lines 367-372 as “… There are some limitations to our analysis that may either arise from the assumptions of the model or its parametrization since we used biologically plausible parameters based on current evidence yet some may be refined as more comprehensive data become available. For example, the COVID-19 induced mortality rate derived for this study based on data from Uganda is relatively lower than global values (e.g., those reported in [33]) and this may be validated in future. …”**

An estimate of 40% asymptotic individuals could hold true, there are multiple studies citing closer to 20% (e.g. Mizumoto et al.), however some more recent studies predict as high as 80% (Day M. “Covid-19: four fifths of cases are asymptomatic, China figures indicate.”). Mentions of such literature should be included.

**We have added a citation in Table 1 of Oran and Topol (2020) on “Prevalence of Asymptomatic SARS-CoV-2 Infection”** https://www.acpjournals.org/doi/pdf/10.7326/M20-3012 **who reported that “Asymptomatic persons seem to account for approximately 40% to 45% of SARS-CoV-2 infections, and they can transmit the virus to others for an extended period, perhaps longer than 14 days”. We also included Baud et al. (2020), Mizumoto et al. (2020), Kirenga et al. (2020), Ing et al. (2020) and Nishiura et al. (2020) to highlight and emphasize the wide variation in reported asymptomatic percentages and we cite these four studies that are based on data in different countries. This is on page 18 in lines 372-375 as “… The other parameter that has been reported to vary widely across countries is the percentage of asymptomatic individuals. Several studies have estimated this proportion using data from different sources including hospitals and cruise ships and their estimated values have ranged from 18% to 81% [34-37]. …”**

Similarly recovery rate, traced-and-isolated rates ( c ), infectivity in hospitals, need closer scrutiny. All model parameters need more source citations.

**Limited data was somehow a hurdle to this analysis and we had to utilize all the then available information from Ministry of health addresses and press releases as well as data available in literature that was applicable to the Ugandan situation whenever possible. Where available, we have added new citations in Table 1 and also added some discussion points on the widely varying parameters.**

**The recovery rate was estimated from Ministry of information which indicated that an admitted patient spent 20 days in hospital on average. This information is in the same range with the data reported in the WHO- China report (see** https://www.who.int/docs/default-source/coronaviruse/who-china-joint-mission-on-covid-19-final-report.pdf ) **in which, using available preliminary data, the median time from onset to clinical recovery for mild cases was approximately 2 weeks and was 3-6 weeks for patients with severe or critical disease. We have added the WHO-China report as a secondary citation for the recovery rate in Table 1.**

**The traced-and-isolated rate (c) was set to 20% based on briefings from the Ministry of Health while addressing challenges in contact tracing due to its labor and other resources intensity as well as the misinformation and intentional refusal by the infected individuals to reveal all their contacts. This number could vary and in a scenario analysis, we assessed to impact of enhancing contact tracing and had (in the original manuscript) written that “…Finally the effect of enhancing contact tracing efforts is modeled through assuming that after 58 days, twice as many latently infected individuals are traced and all infectious periods are only one day…” However, now that we are trimming the content of the manuscript, that content is unfortunately omitted and only the sensitivity analysis has information on the potential impact of contact tracing magnitude on the outcomes.**

**On infectivity in hospitals, note that Uganda took more than 2 months to register a COVID-19 case in frontline health workers. The first case among health workers was registered in the last week of May 2020** https://www.newvision.co.ug/news/1526183/covid-19-senior-epidemiologist-dies**) and by the time of our analysis, the number of hospital-acquired infections was nearly negligible. However, we assessed the impact of having hospital acquired infections as part of the sensitivity analysis (still in the manuscript) and also in a scenario analysis (which has since been omitted following the recommendations).**

Parameter q can be removed if it is not used in the model.

**This comment is appreciated but we thought that, for model flexibility, completeness and robustness, we should maintain the parameter q in the ode system. However, we have improved on the assumption to add more justification on page 8 in lines 155-158 as “… (h) Although viral loads have been reported to be similar between asymptomatic and symptomatic patients [30], the asymptomatically infected individuals may have a reduced infectivity because they may not cough or sneeze as much as the symptomatic. This however needs to be further elucidated. …”**

Moving on to the model itself, a major concern is that individuals who are contacted via test-and-trace are moved to the hospitalisation class (If I understood correctly). This is a major oversight as it will be a significant move of individuals, having a considerable impact on the resulting dynamics, and invalidating any assessment of total hospitalisation numbers (such as figures 2,3,5). This estimation of hospitalised individuals is a major use of these models, and so the assessment must be rigorous.

**Yes, you understood the transition correctly as was regrettably originally (poorly) written.**

**We had missed a critical component describing the transition from E directly to H. We had not explicitly mentioned the fact that traced E individuals can only transit to H after completing their latent period. The proportion c is first isolated in hospital-like isolation settings until they test positive. By doing this, their infectious period is technically reduced to zero and are thus denied a chance of infecting others in the absence of hospital-acquired infections.**

**We have now improved the write up on this assumption to capture this key concept on page 7 in lines 134-139 to “… (b) Upon infection, some latently infected individuals can be identified (e.g., through contact tracing) and subsequently individually isolated under high biosecurity conditions leading to their eventual hospitalization upon testing positive (i.e. completing their latent period), thereby denying them a chance of ever infecting other individuals in the community, while others may remain in the community and become infectious up until when they are identified and hospitalized. …”.**

**Technically, from the transition term from E to H occurring at a rate** $\boldsymbol{c\rho E}$**, it can be seen that the proportion c of the latently infected individuals still first go through their latent period before transiting to the H compartment and hence this transition has no obvious effect on the predicted number of hospitalized. For model parsimony, we did not include a separate isolation compartment since there was no intension to draw conclusions on the dynamics in the isolated compartment. However, even without that compartment, the alternative formulation is robust and it captures all the key processes and it carters for the latent period of the traced E individuals. Hence we believe the formulation used does not affect the dynamics in the H compartment.**

**Practically, the transition from latently infected compartment directly to the hospitalized compartment as modeled was intended to mimic the reality on ground where, once identified as contacts, individuals were individually isolated in designated centers with no possibility of mingling with other (susceptible or otherwise) individuals besides health workers who are always protected with PPE. These individuals were regularly tested for COVID-19 and moved to treatment centers upon testing positive. This nature of isolation mimicked the settings in hospital in that the individuals in isolation that later turned out to have been latently infected by the time of their tracing did not get a chance to infect other individuals (outside hospital-acquired infections) upon becoming infectious.**

I am also sceptical of the implementation of imported cases. Having this influx of individuals (and I don’t fully understand how these imported cases subsequently leave the population pool in an equal amount) be dependent on the host population size (N) doesn’t make sense. In general, one could perhaps argue for a constant population (in/out) from the infected class, but this formulation is not suitable for then drawing conclusions on the dynamic impact of imported cases from neighbouring countries. It would be better to develop a model of multiple interacting SEIHR networks for this. I would recommend either removing this aspect or better framing this as “the dynamic impact of constant external disease pressure”.

**On the implementation of imported cases as a function of host population size, we solely based this formulation on the fact that the demand for goods that the arriving truck drivers deliver would likely be directly proportional to the population size i.e. the demand and supply dynamics of economics. Since the timeframe over which the predictions were to be made was so short, we opted for the epidemic model in which we excluded the births and natural mortality. Over this short prediction period, we expect that, under the prevailing circumstances, the host population size may slightly fluctuate and hence dN/dt≠0.**

**On the suggestion to develop a model of multiple interacting SEIHR networks, we foresee a big hurdle relating to model parameterization and generally acquisition of outbreak data from Kenya, Tanzania (that officially already declared COVID-19 a hoax), South Sudan and Rwanda (that is under political tension with Uganda). The only readily available data was the number of arriving truck drivers (infected or not) that the Ministry of Health-Uganda releases daily based on daily testing at the border posts. It is likely that information derived from the testing data at the border posts may not be fully reflective of the disease dynamics in the neighboring countries. However, due to data limitations, we used that information in the current modeling framework.**

**The transient dynamics of the disease are happening simultaneously within the East African region. However, our model only incorporated the dynamism for the disease dynamics in Uganda and assumed a constant external disease pressure. We note that the coupling of the disease dynamics in Uganda with those in the neighboring through the arrival of infected truck drivers is partly governed by the disease prevalence in those countries. However, it is hard to get a grip on the required data from some of those countries to substantiate the network approach.**

**Therefore, in line with your (greatly appreciated) technical advice on modeling, we have opted to reframe this particular analysis’ header on page 11 in line 228 and the section header for 4.3 on page 14 in line 283 as “… The dynamic impact of constant external disease pressure …”. We have also added a discussion point to this effect on page 18 in lines 376-380 as “… Given the demonstrated coupling of Uganda’s disease dynamics with those in her neighboring countries, network based modeling approaches would be better suited to assess potential impact of the coupling pathways on the disease dynamics. Although we assumed constant external disease pressure in this study, we recommend that future studies adopt the network modeling approach whenever relevant data to substantiate those network based models is available. …”**

I would also be interested in more information on the exact lockdown rules in Uganda to defend the choice of lockdown modelling. The authors have a percentage of the population which is made impossible to become infected due to lockdown. This is appropriate for strict lockdown systems, such as that in Cyprus, where individuals may not leave home without permission, but for systems such as the UK where individuals may leave home freely for essential trips, a far reduced transmission rate may be more appropriate than a flat impossibility of transmission (e.g. Rawson et al. “How and when to end the COVID-19 lockdown”).

# Our choice of lockdown modelling in which we assumed that a percentage of the population was impossible to become infected due to lockdown was motivated by level of strictness in implementing lockdown restrictions in Uganda which was one of the strictest in Africa. Content to this effect can be found in an article whose link is indicated from which I quote the following; “… In March, Uganda introduced one of the most stringent lockdowns in Africa, banning cars and public gatherings, shutting down shopping centres, places of worship, schools and entertainment centres, and putting in place a night-time curfew.” (See <https://www.telegraph.co.uk/global-health/science-and-disease/ugandas-tough-approach-covid-19-hurting-citizens/> ). There were roadblocks manned by Military Police, the Army and Local defense personnel to ensure compliance. The measures were so strict to the extent of shooting some noncompliant citizens. Some of those acts are reflected in the BBC news article titled “Uganda - where security forces may be more deadly than coronavirus” (see <https://www.bbc.com/news/world-africa-53450850> )

**The approach of Rawson et al. 2020 in which the population was split into a quarantine and a non-quarantined group differing by the rate of virus transmission and the groups being connected by the modeled release strategies from lockdown seems to be one of the most suitable approaches for the countries in which lockdown restrictions were less strict.**

**We have added a justification for our choice of lockdown modeling in the discussion on page 18 in lines 380-386 as “On lockdown modeling, we assumed that a percentage of the susceptible population was isolated by the lockdown restrictions and hence impossible to become infected. This assumption is justified by the strictness in enforcing these measures in Uganda. However, for relatively lax implementation of the measures, alternative approaches such as that involving splitting the population into the quarantine and non-quarantine groups as done by [Rawson et al. 2020] could be more suitable.**”

When the model is introduced (from L102), it is very difficult to interpret the model initially, needing to jump between table 1, figure 1, equations (1)-(5), and brief paragraphs. I would instead urge presenting each equation, one-by-one, and explaining specifically each term (and parameter) in each equation. This may seem excessive, but makes interpreting a model considerably easier.

**The flow of content in this section has been improved up by numbering and then describing each equation fully in the text right below where all the equations appear. The content is capture on page 9 in lines 179-200 as “In Equation (3.1), the term** $\left( \boldsymbol{1-}\left( \boldsymbol{a+e} \right) \right)\boldsymbol{\lambda N}$ **represents the number of arriving susceptible individuals per day,** $\frac{\boldsymbol{\beta bS}\left( \boldsymbol{q}\boldsymbol{I}_{\boldsymbol{a}}\boldsymbol{+}\boldsymbol{I}_{\boldsymbol{s}}\boldsymbol{+gH} \right)}{\boldsymbol{N}}$ **represents the number of susceptible individuals that become latently infected per day,** $\boldsymbol{\tau R}$ **represents the number of previously immune individuals that lose their disease-induced immunity to become susceptible per day and** $\boldsymbol{\lambda S}$ **represents the number of departing susceptible individuals per day. In Equation (3.2), the term** $\boldsymbol{e\lambda N}$ **represents the number of arriving latently infected individuals per day while** $\boldsymbol{\rho E}$ **represents the number of latently infected individuals that progress to become infectious per day. In Equation (3.3), the term** $\boldsymbol{a\lambda N}$ **represents the number of arriving asymptomatically infectious individuals per day,** $\boldsymbol{r\rho E}$ **represents the number of latently infected individuals that become asymptomatically infectious per day and** $\boldsymbol{\omega}_{\boldsymbol{a}}\boldsymbol{I}_{\boldsymbol{a}}$ **represents the number of asymptomatically infectious individuals that are hospitalized per day. In Equation (3.4), the term** $\left( \boldsymbol{1-}\left( \boldsymbol{c+r} \right) \right)\boldsymbol{\rho E}$ **represents the number of latently infected individuals that become symptomatically infectious per day,** $\boldsymbol{\sigma}_{\boldsymbol{s}}\boldsymbol{I}_{\boldsymbol{s}}$ **represents the number of symptomatically infectious individuals that succumb to the disease before being hospitalized per day and** $\boldsymbol{\omega}_{\boldsymbol{s}}\boldsymbol{I}_{\boldsymbol{s}}$ **represents the number of symptomatically infectious individuals that are hospitalized per day. In Equation (3.5), the term** $\boldsymbol{c\rho E}$ **represents the number of latently infected individuals that were traced and isolated prior to being hospitalized upon testing positive per day,** $\boldsymbol{\omega}_{\boldsymbol{a}}\boldsymbol{I}_{\boldsymbol{a}}$ **represents the number of asymptomatically infectious individuals that are hospitalized per day,** $\boldsymbol{\omega}_{\boldsymbol{s}}\boldsymbol{I}_{\boldsymbol{s}}$ **represents the number of symptomatically infectious individuals that are hospitalized per day,** $\boldsymbol{\sigma}_{\boldsymbol{h}}\boldsymbol{H}$ **represents the number of hospitalized individuals that succumb to the disease per day while** $\boldsymbol{\alpha H}$ **represents the number of hospitalized individuals that recover from the disease per day. Lastly, in Equation (3.6), the term** $\boldsymbol{\lambda R}$ **represents the number of departing recovered individuals per day and the other two terms are described above.”**

The above issues then raise concern with the interpretation of results:

**It is true that, if not well clarified as was the case in the earlier version of the manuscript, doubt would be cast on the interpretation of the results. However, we hope that the explanations given, amendments and clarifications made will eliminate (some of) the original doubt.**

4.2 – This section seems the most powerful to focus on, and could be expanded by comparing and fitting to Ugandan case data.

**The model was fitted to Uganda’s case data for the first 58 days since the confirmation of the first case (when this analysis commenced) and the figure is below. The figure was helpful in assessing our model biological plausibility during parametrization.**

The current primary result is not particularly useful or enlightening, less people in lockdown = more people infected. More interesting would be to then expand this to ask “how strict does a lockdown need to be to not exceed hospital capacity?” “When should a lockdown be ended?” etc. The current metric of hospitalisations is incorrect due to the above test-and-trace classification issues.

**We agree that there could be other useful results from this study and we also acknowledge that we had not clearly explained how we implemented the trace-test-and- hospitalize in latently infected class, and this could have partly led to your doubting of the hospitalization metric. We have now addressed the latter while addressing your previous comment above and also added more content on this at the end of the rebuttal to this very point. We hope that the amendments and explanations given on this matter suffice.**

**One of the primary aims of the study was to inform policy on how to release lockdown restrictions i.e., under a given percentage of susceptible individuals, when will the then 3200 hospital capacity. On this, we report on page 14 lines 278-281 as “… These results demonstrate that if we ease lockdown by releasing 50% of susceptible population for the Ugandan situation with 3200 beds and not all are of ICU-like capacity, within 100 days the COVID-19 related hospitalization demand would have already overwhelmed the current resources. …” This is in line with your opinion that we frame the study to address the question; “how strict does a lockdown need to be to not exceed hospital capacity?”.**

**Additionally, on using the study to answer the question; “When should a lockdown be ended?”, we address a closely related question in section 4.4 on the possibility of a second wave on page 15 in line 297. In that analysis, we determine when the government can let up to 75% of the population out of lockdown while mitigating the second wave possibility. We report on page 15 in lines 302-304 as “… The simulations reveal that with the current levels of contact tracing, having released up to 75% of susceptible after 150 days would guarantee emergence of a second epidemic wave. …” and also in Figure 4 caption on page 27 lines 547-551 “… Panel (a) shows easing of lockdown from 10% to 30% susceptible after 58 days and further easing to 75% after 210 days of lockdown and could be sooner if contact tracing efforts were intensified. Panel (b) is a case of easing lockdown from 10% to 30% after 58 days followed by 75% susceptible after 150 days of lockdown – a case of too many and too soon. …”**

**On the point of a possible incorrectness of the hospitalization metric that your raise, we think that that is rectified by the fact that the traced and tested latently infected individuals will on join the Hospitalized compartment upon completing their latent period (since there are first individually isolated until testing positive after which they are taken to hospital). We believe that this alternative formulation still captures the dynamics appropriately since it does not in any way reduce on the latent period. The only difference that this alternative formulation correctly captures is the elimination of the infectious potential for the traced and isolated latently infected individuals since they are not accorded a chance to mix with the susceptible individuals upon becoming infectious; they will be hospitalized straightaway.**

4.3 – I currently don’t think this model formulation is powerful enough to answer questions on the impact of imported cases. The result that “COVID remains endemic” is due precisely to the model formulation. The constant influx of infection from these cases ensures an endemic outbreak. If the authors wanted to investigate the impact of imported cases then this influx should be variable. Then the model can answer questions such as “When should imports from country A be allowed based on their case rates?”.

**Agreed!**

**We have eliminated the subsection detailing the analyses of “*The impact of hospital acquired infections, regional disease prevalence and contact tracing efforts”.* Former subsection 4.5 and Figure 4.5 have been omitted from the revised manuscript. *We also* revised the results and discussion accordingly. The results on potential impact of varying contact tracing effort are now only discussed in line with the sensitivity analysis. For example, in the results on page 15 in lines 314-319 as “… From the sensitivity analysis, the most impactful parameters identified include the arriving proportion of infected individuals** $\boldsymbol{(a)}$**, the per capita recruitment rate** $\boldsymbol{(\lambda)}$**, the disease transmission rate** $\boldsymbol{(}\boldsymbol{\beta}\boldsymbol{)}$**, the proportion of susceptible population available for infection** $\boldsymbol{(b)}$**, and the efforts of contact tracing and subsequent isolation of latently infected individuals** $\boldsymbol{(c)}$ **among others. …” Otherwise, these parameters only appear at their default values in the other analyses. The potential impact of varying hospital acquired infections is now discussed on page 18 in lines 360-365 as “… Although the parameter influencing the number of hospital acquired infections was of relatively low impact on the number of symptomatic cases in the sensitivity analysis, field experience with handling COVID-19 in other countries (note that Uganda did not report any hospital acquired infection for the first two months of her epidemic) as well as diseases such as Ebola in Uganda dictates that this should be maintained at a bare minimum to ensure that health workers do not shun their duties. …”**

4.4 – This section will be insightful, however the above concerns on model formulation and parameterisation need to be addressed first.

**Our original shallowness in explaining some of the key approaches and assumptions (such as that on the trace-test-and-Hospitalize a fraction of the latently infected individuals) in our model could have led to most of the queries raised. We have improved the write up as described under each of queries raised above. We hope that all or most of the concerns have now been addressed and we also hope that the amendments and rebuttals are helpful in generating more insights on our approaches.**

4.5 – I don’t think the model is suitable for assessing the impact of hospital-acquired infections currently. This is an interesting dynamic, but would benefit from a more bespoke model, with separate healthcare compartmentalisations. The current hopsitalisation framework (see above) invalidates the current results.

**Following your recommendation, we have eliminated this subsection and refocused the manuscript accordingly. The originally assessed parameters are now only mentioned in the sensitivity analysis results e.g., on page 15 in lines 314-318 as “… From the sensitivity analysis, the most impactful parameters identified include the arriving proportion of infected individuals** $\boldsymbol{(a)}$**, the per capita recruitment rate** $\boldsymbol{(\lambda)}$**, the disease transmission rate** $\boldsymbol{(}\boldsymbol{\beta}\boldsymbol{)}$**, the proportion of susceptible population available for infection** $\boldsymbol{(b)}$ **and the efforts of contact tracing and subsequent isolation of latently infected individuals** $\boldsymbol{(c)}$ **among others. …”**

4.6 – I’m not sure the text here actually correlates with the sensitivity analysis of Figure 6. The text states that a, e, b, and g are most influential. However Figure 6 seems to infer that a and lambda are the most impactful, and the others have minor impact. Certainly g and e seem of low impact. Also, the bins of Figure 6 should be marked with their respective parameters. The current colour scheme is hard to distinguish.

**This error has been rectified on page 15 in lines 314-318 as “… From the sensitivity analysis, the most impactful parameters identified include the arriving proportion of infected individuals** $\boldsymbol{(a)}$**, the per capita recruitment rate** $\boldsymbol{(\lambda)}$**, the disease transmission rate** $\boldsymbol{(}\boldsymbol{\beta}\boldsymbol{)}$**, proportion of susceptible population available for infection** $\boldsymbol{(b)}$ **and the efforts of contact tracing and subsequent isolation of latently infected individuals** $\boldsymbol{(c)}$ **among others. …”.**

**The Figure (now #5) identifies the parameters using the legend on the right in the order of appearance of the bars in the graph with the topmost entry in the legend corresponding to the first bar in the graph. The figure caption on page 28 in lines 556-562 has be improved to explicitly explain this as “…Figure 5. Pearson’s Rank Correlation Coefficients (PRCCs) tornado simulation of the model’s most sensitive parameters sampled 300 times using the Latin hypercube sampling scheme. Sensitivity variation with respect to time is analyzed at two time points, 30 days and 58 days that could be used as initialization points or start times for the intervention measures. The order of appearance of parameters in the figure legend follow the left to right order in labeling the corresponding bars. For example, the topmost label corresponds to the first bar in the figure and so on. …”**

Minor Comments:
L51/L52 – “measures reduced the susceptible population to about 10%”, this is important, is there a citation for this?

**This was mentioned in press briefings by the Ministry of Health and other non-documented outlets. However, it can be approximated based on the work demographic data of Uganda (Source: Uganda Bureau of Statistics) where 80% of Ugandan’s workforce is employed in the non-formal sector and hence were less likely to have been categorized as “Essential workers” as per the definition used during lockdown. Only a few market vendors (who were not even allowed to return home to their families) as well as essential, health service providers and essential cargo transporters were allowed to operate. Also of the 43 million Ugandans, 15 million are school going and hence congregate in schools yet all schools were closed during lockdown.**

L129 – Does this mean that asymptomatic individuals are hospitalised? I assume this is due to the test-and-trace, this needs to be changed.

**Yes. Everyone that tested positive- symptomatic or not- had to be hospitalized to prevent them from spreading the disease in the community. This went on until recently (as recent as early November 2020) when the hospitals got overwhelmed.**

L266 – What is meant by undetected infectious here? Mentioned specifically which model variable you mean when referenced (e.g. I/E)

**The undetected infectious individuals referred to there and throughout the manuscript comprise of the yet-to-be hospitalized asymptomatic and sympotomatic infectious individuals. We have explicitly defined this concept on its first appearance in the manuscript on page 14 in lines 277-278 as “…the undetected infectious individuals (comprising of the yet-to-be hospitalized asymptomatic and symptomatic infectious individuals)…”**

The model is good and I think it is of critical importance to investigate country-specific disease trajectories, the scope and details of the model just need to be refined. I look forward to seeing a more refined revision and believe it will make a strong contribution when finished.

**This encouraging comment as well as the suggested amendments are greatly appreciated.**
