# Supplementary material for: Mathematical modeling of COVID-19 transmission dynamics in Uganda: Implications of complacency and early easing of lockdown
Source: PLoS One. 2021 Feb 22;16(2):e0247456. doi: 10.1371/journal.pone.0247456 (PMC7899325; doi:10.1371/journal.pone.0247456)
Supplement: Attachment — Submitted filename: Ugandan Model - 2nd Reviewer Comments.docx [file pone.0247456.s005.docx]

Initial Reviewer Comments

Author Responses

Reviewer Responses

Reviewer #3: The manuscript utilises a compartmental SEIHR framework to assess the disease dynamics in Uganda, and consider how to tackle future infection possibilities. Current manuscript revisions have already improved the manuscript well. While I feel the work has great potential to advance the understanding of disease dynamics within Uganda, I have substantial concerns with the current model formulation, and the conclusions the authors draw.

The primary issue is that the authors attempt to, arguably, do too much with the model, and in doing so each conclusion suffers. I list below my general concerns with the model formulation, and which scenarios are ill-posed for the current model. In general I would urge the authors to restrict their scope, and tweak the model to draw stronger conclusions on a specific research question or two. I feel the model may be best suited with minor adjustment to assessing “The possibility of a second wave infection”, or “The impact of lockdown measures”, while it is currently not an appropriate model for assessing “The impact of magnitude of imported cases” or “Impact of hospital acquired infections”.
The Introduction is very strong and suitably frames the work.

We agree that trimming and refocusing the content tremendously improves that quality of the manuscript and as suggested by the reviewer, the manuscript has been refocused to address three specific questions namely; 1) assessing the potential impact of the implemented lockdown measures, 2) the dynamic impact of constant external disease pressure (phrasing suggested by the reviewer in the later comments), and 3) the possibility of the second wave of infection. We also maintain the section on sensitivity analysis.

I thank the authors for the time taken to address my concerns. The manuscript is hugely improved and presents a very strong and interesting model investigating the specific mitigation strategies and disease-trajectory within Uganda. I must however request some minor additional adjustments before I can finally recommend for publication, primarily regarding the justification of some model parameters. Justification for these is discussed in the below responses of the authors, however this needs to also be conveyed in the manuscript itself. I address these specific points directly below, and summarise at the end. Apologies to further slow the process but these model parameters must be well-explained for readers, I appreciate this may require a re-plotting of some figures and results presentation, which is always frustrating, but I think it is very important for the final presentation of results.

General Concerns
My initial concerns are with the model parameters (Table 1). Many parameters cite the Ugandan data portal (reference [4]) for their values, however I cannot locate these model parameters, nor can I infer them from Ugandan case data.

For reliability of the study outcomes in informing Uganda policy interventions to manage the disease, it was necessary that the study uses localized parameters whenever possible. However, as was the case with other earlier modeling studies, only scanty information about the pathogen and the disease in general was available and more is just being generated as the pandemic progresses. Therefore, some of the earlier simulation studies utilized the limited data together with sensitivity analyses to infer disease dynamics under varying intervention scenarios. It is hoped that as more knowledge about the pathogen and its transmission pathways in generated, the models will be updated accordingly.
For our Ugandan case, not all required parameters have been documented in citable literature and this was a hurdle to the current study. For example, besides the number of new cases, recoveries and deaths that are mandatorily documented and forwarded to WHO among other stakeholders, the other parameters that cite the Ministry of Health data (reference 4) are mainly inferred or derived from the routine addresses by the personnel from the Ministry of Health, Uganda virus research institute and the office of the presidency among others and are based on observational data. Examples of such include the arrival rate of truck drivers (both infected and non-infected), number of returning residents through airports, time spent in hospital for the admitted cases which partly informs the model recovery rate, the proportion of asymptomatic infected individuals etc. In our parameter estimation, content from these routine national addresses and press releases which is often summarized in news outlets was used to infer some of the model parameters. One of such briefings can be found online at https://www.health.go.ug/document/update-on-covid-19-response-in-uganda/ and https://www.health.go.ug/covid/category/press-release/ Studies are being conducted and it is hoped that such new studies will have data from which other more precise parameters can be obtained. For example the recently published Kirenga et al. (2020) “Characteristics and outcomes of admitted patients infected with SARSCoV-2 in Uganda” https://www.ncbi.nlm.nih.gov/pmc/articles/PMC7477797/pdf/bmjresp-2020-000646.pdf validates some of the parameter estimates.
We have checked the literature again and identified some articles that we could cite for some of the parameters in Table 1. For recruitment rate [Ministry of Health -Uganda. UPDATE ON THE COVID-19 OUTBREAK IN UGANDA available online at https://www.health.go.ug/covid/category/press-release/ accessed on 1 December 2020. 2020.], for recovery rate of hospitalized individuals [WHO-China. Report of the WHO-China Joint Mission on Coronavirus Disease 2019, available online at https://www.who.int/docs/default-source/coronaviruse/who-china-joint-mission-on-covid-19-final-report.pdf, accessed 3 June 20202020.] and for percentage of latently infected individuals in community that becomes asymptomatic [Daniel P. Oran A, Eric J. Topol. Prevalence of Asymptomatic SARS-CoV-2 Infection. Annals of Internal Medicine. 2020;173(5):362-7. doi: 10.7326/m20-3012 %m 32491919.]

These are good additions. I of course appreciate the difficulties in ascertaining specific model parameters, and do not take issue with “back-of-the-envelope” calculations from case data. Rather it just needs to be made clear which parameters are rougher estimates and which are not.

Some parameters are also very far from similar parameter estimates in the literature. For example, a disease-induced mortality rate of 0.001 is far low than estimates elsewhere in the literature. The hospital mortality rate of 0.0001 is even more peculiar. See for example, Baud et al. (2020) “Real estimates of mortality following COVID-19 infection.”.

The low disease-induced death rate that used in our study is for the Ugandan situation where Uganda did not register any COVID-19 related death in the first four months from the first case (i.e. March 22nd to July 22nd 2020). The first death was registered on 23rd July 2020 and at that point, the cumulative number of cases was 1079 with 971 recoveries. In other countries, using data as of March 1st 2020 (i.e. approx. 4 months (December to March) into the outbreak), Baud et al. (2020) reported mortality rate of 3·6% (95% CI 3·5–3·7) in China and 1·5% [1·2–1·7] outside of China and when adjusted for incubation period delay, mortality rates would be 5·6% (95% CI 5·4–5·8) for China and 15·2% (12·5–17·9) outside of China. Moreover, even as of December 1st 2020, globally there are approximately 63.7m cases, 1.5m deaths and 44.1m recoveries while in Uganda, there have been 20,459 cases, with 205 fatalities and 8989 recoveries. These numbers may crudely indicate both the case-fatality ratio and Recovered-fatality ratio (obtained from considering only the resolved cases) are relatively lower in Uganda.
For completeness and clarity, we have added the following “justification” also citing Baud et al (2020) in the discussion on page 18 in lines 367-372 as “… There are some limitations to our analysis that may either arise from the assumptions of the model or its parametrization since we used biologically plausible parameters based on current evidence yet some may be refined as more comprehensive data become available. For example, the COVID-19 induced mortality rate derived for this study based on data from Uganda is relatively lower than global values (e.g., those reported in [33]) and this may be validated in future. …”

Following the quoted numbers of 205 deaths and 20,459 cases, this equates to a mortality rate of ~0.01, as opposed to either 0.001 or 0.0001 as used in the manuscript. I understand then that the authors have based their mortality rates on the 1 out of 1079 cases from the initial outbreak period. I think it is however clear to see as testing has increased that this initial amount was not indicative of the true mortality rate. Some parameter inaccuracy is of course permissible given how much is still unknown, and I of course encourage using data specific to Uganda, however the values used show signs of being out by a factor of 10 or 100. This will also directly impact the number of hospitalisations observed. I am comfortable with a general difference in mortality between hospitalised and non-hospitalised, however 0.0001 is just a bit too far to my mind! In general, something like multiplying both these factors by 10 (0.01 and 0.001 respectively) seems a more sensible bet, and closer to the true mortality rate witnessed in Uganda.

An estimate of 40% asymptotic individuals could hold true, there are multiple studies citing closer to 20% (e.g. Mizumoto et al.), however some more recent studies predict as high as 80% (Day M. “Covid-19: four fifths of cases are asymptomatic, China figures indicate.”). Mentions of such literature should be included.

We have added a citation in Table 1 of Oran and Topol (2020) on “Prevalence of Asymptomatic SARS-CoV-2 Infection” https://www.acpjournals.org/doi/pdf/10.7326/M20-3012 who reported that “Asymptomatic persons seem to account for approximately 40% to 45% of SARS-CoV-2 infections, and they can transmit the virus to others for an extended period, perhaps longer than 14 days”. We also included Baud et al. (2020), Mizumoto et al. (2020), Kirenga et al. (2020), Ing et al. (2020) and Nishiura et al. (2020) to highlight and emphasize the wide variation in reported asymptomatic percentages and we cite these four studies that are based on data in different countries. This is on page 18 in lines 372-375 as “… The other parameter that has been reported to vary widely across countries is the percentage of asymptomatic individuals. Several studies have estimated this proportion using data from different sources including hospitals and cruise ships and their estimated values have ranged from 18% to 81% [34-37]. …”

Similarly recovery rate, traced-and-isolated rates ( c ), infectivity in hospitals, need closer scrutiny. All model parameters need more source citations.
Limited data was somehow a hurdle to this analysis and we had to utilize all the then available information from Ministry of health addresses and press releases as well as data available in literature that was applicable to the Ugandan situation whenever possible. Where available, we have added new citations in Table 1 and also added some discussion points on the widely varying parameters.
The recovery rate was estimated from Ministry of information which indicated that an admitted patient spent 20 days in hospital on average. This information is in the same range with the data reported in the WHO- China report (see https://www.who.int/docs/default-source/coronaviruse/who-china-joint-mission-on-covid-19-final-report.pdf ) in which, using available preliminary data, the median time from onset to clinical recovery for mild cases was approximately 2 weeks and was 3-6 weeks for patients with severe or critical disease. We have added the WHO-China report as a secondary citation for the recovery rate in Table 1.
The traced-and-isolated rate (c) was set to 20% based on briefings from the Ministry of Health while addressing challenges in contact tracing due to its labor and other resources intensity as well as the misinformation and intentional refusal by the infected individuals to reveal all their contacts. This number could vary and in a scenario analysis, we assessed to impact of enhancing contact tracing and had (in the original manuscript) written that “…Finally the effect of enhancing contact tracing efforts is modeled through assuming that after 58 days, twice as many latently infected individuals are traced and all infectious periods are only one day…” However, now that we are trimming the content of the manuscript, that content is unfortunately omitted and only the sensitivity analysis has information on the potential impact of contact tracing magnitude on the outcomes.
On infectivity in hospitals, note that Uganda took more than 2 months to register a COVID-19 case in frontline health workers. The first case among health workers was registered in the last week of May 2020 https://www.newvision.co.ug/news/1526183/covid-19-senior-epidemiologist-dies) and by the time of our analysis, the number of hospital-acquired infections was nearly negligible. However, we assessed the impact of having hospital acquired infections as part of the sensitivity analysis (still in the manuscript) and also in a scenario analysis (which has since been omitted following the recommendations).

These are good justifications, and as such need to be included in the manuscript! I appreciate this is quite a lot of text to include, so I think my best advice would be to create a separate appendix or supplementary file that directly explains the inference of all these model parameters from case data and ministerial reports / press-releases.

Parameter q can be removed if it is not used in the model.

This comment is appreciated but we thought that, for model flexibility, completeness and robustness, we should maintain the parameter q in the ode system. However, we have improved on the assumption to add more justification on page 8 in lines 155-158 as “… (h) Although viral loads have been reported to be similar between asymptomatic and symptomatic patients [30], the asymptomatically infected individuals may have a reduced infectivity because they may not cough or sneeze as much as the symptomatic. This however needs to be further elucidated. …”

This is a good idea.

Moving on to the model itself, a major concern is that individuals who are contacted via test-and-trace are moved to the hospitalisation class (If I understood correctly). This is a major oversight as it will be a significant move of individuals, having a considerable impact on the resulting dynamics, and invalidating any assessment of total hospitalisation numbers (such as figures 2,3,5). This estimation of hospitalised individuals is a major use of these models, and so the assessment must be rigorous.

Yes, you understood the transition correctly as was regrettably originally (poorly) written.
We had missed a critical component describing the transition from E directly to H. We had not explicitly mentioned the fact that traced E individuals can only transit to H after completing their latent period. The proportion c is first isolated in hospital-like isolation settings until they test positive. By doing this, their infectious period is technically reduced to zero and are thus denied a chance of infecting others in the absence of hospital-acquired infections.
We have now improved the write up on this assumption to capture this key concept on page 7 in lines 134-139 to “… (b) Upon infection, some latently infected individuals can be identified (e.g., through contact tracing) and subsequently individually isolated under high biosecurity conditions leading to their eventual hospitalization upon testing positive (i.e. completing their latent period), thereby denying them a chance of ever infecting other individuals in the community, while others may remain in the community and become infectious up until when they are identified and hospitalized. …”.
Technically, from the transition term from E to H occurring at a rate cρE, it can be seen that the proportion c of the latently infected individuals still first go through their latent period before transiting to the H compartment and hence this transition has no obvious effect on the predicted number of hospitalized. For model parsimony, we did not include a separate isolation compartment since there was no intension to draw conclusions on the dynamics in the isolated compartment. However, even without that compartment, the alternative formulation is robust and it captures all the key processes and it carters for the latent period of the traced E individuals. Hence we believe the formulation used does not affect the dynamics in the H compartment.
Practically, the transition from latently infected compartment directly to the hospitalized compartment as modeled was intended to mimic the reality on ground where, once identified as contacts, individuals were individually isolated in designated centers with no possibility of mingling with other (susceptible or otherwise) individuals besides health workers who are always protected with PPE. These individuals were regularly tested for COVID-19 and moved to treatment centers upon testing positive. This nature of isolation mimicked the settings in hospital in that the individuals in isolation that later turned out to have been latently infected by the time of their tracing did not get a chance to infect other individuals (outside hospital-acquired infections) upon becoming infectious.

My apologies to the authors, as I was not aware of the specific isolation protocol within Uganda. Indeed, having learnt of this, it strengthens my belief that this work will make a strong contribution as a study of country-specific protocol. I might even recommend adding a brief paragraph to the introduction explicitly stating the use and protocol of isolation centres, not just to defend the modelling choices, but to stress the importance of this work. I found this document to be very useful in understanding the procedures: <https://www.health.go.ug/covid/wp-content/uploads/2020/04/Updated-Guide-Draft_24April20_CLEAN.pdf> and would recommend perhaps citing this to help explain to readers as naïve as I was! I might also suggest adding somewhere in the abstract how this model captures “specific isolation protocol followed in Uganda”, as I think this is a major strength of the model that should be advertised.

I am also sceptical of the implementation of imported cases. Having this influx of individuals (and I don’t fully understand how these imported cases subsequently leave the population pool in an equal amount) be dependent on the host population size (N) doesn’t make sense. In general, one could perhaps argue for a constant population (in/out) from the infected class, but this formulation is not suitable for then drawing conclusions on the dynamic impact of imported cases from neighbouring countries. It would be better to develop a model of multiple interacting SEIHR networks for this. I would recommend either removing this aspect or better framing this as “the dynamic impact of constant external disease pressure”.

On the implementation of imported cases as a function of host population size, we solely based this formulation on the fact that the demand for goods that the arriving truck drivers deliver would likely be directly proportional to the population size i.e. the demand and supply dynamics of economics. Since the timeframe over which the predictions were to be made was so short, we opted for the epidemic model in which we excluded the births and natural mortality. Over this short prediction period, we expect that, under the prevailing circumstances, the host population size may slightly fluctuate and hence dN/dt≠0.
On the suggestion to develop a model of multiple interacting SEIHR networks, we foresee a big hurdle relating to model parameterization and generally acquisition of outbreak data from Kenya, Tanzania (that officially already declared COVID-19 a hoax), South Sudan and Rwanda (that is under political tension with Uganda). The only readily available data was the number of arriving truck drivers (infected or not) that the Ministry of Health-Uganda releases daily based on daily testing at the border posts. It is likely that information derived from the testing data at the border posts may not be fully reflective of the disease dynamics in the neighboring countries. However, due to data limitations, we used that information in the current modeling framework.
The transient dynamics of the disease are happening simultaneously within the East African region. However, our model only incorporated the dynamism for the disease dynamics in Uganda and assumed a constant external disease pressure. We note that the coupling of the disease dynamics in Uganda with those in the neighboring through the arrival of infected truck drivers is partly governed by the disease prevalence in those countries. However, it is hard to get a grip on the required data from some of those countries to substantiate the network approach.
Therefore, in line with your (greatly appreciated) technical advice on modeling, we have opted to reframe this particular analysis’ header on page 11 in line 228 and the section header for 4.3 on page 14 in line 283 as “… The dynamic impact of constant external disease pressure …”. We have also added a discussion point to this effect on page 18 in lines 376-380 as “… Given the demonstrated coupling of Uganda’s disease dynamics with those in her neighboring countries, network based modeling approaches would be better suited to assess potential impact of the coupling pathways on the disease dynamics. Although we assumed constant external disease pressure in this study, we recommend that future studies adopt the network modeling approach whenever relevant data to substantiate those network based models is available. …”

I’m still a little uncertain as to the accuracy of how in/out-flux of travel from other countries is modelled. However this should not impact the results, so I’m happy for it to be included as it currently is.

I would also be interested in more information on the exact lockdown rules in Uganda to defend the choice of lockdown modelling. The authors have a percentage of the population which is made impossible to become infected due to lockdown. This is appropriate for strict lockdown systems, such as that in Cyprus, where individuals may not leave home without permission, but for systems such as the UK where individuals may leave home freely for essential trips, a far reduced transmission rate may be more appropriate than a flat impossibility of transmission (e.g. Rawson et al. “How and when to end the COVID-19 lockdown”).

Our choice of lockdown modelling in which we assumed that a percentage of the population was impossible to become infected due to lockdown was motivated by level of strictness in implementing lockdown restrictions in Uganda which was one of the strictest in Africa. Content to this effect can be found in an article whose link is indicated from which I quote the following; “… In March, Uganda introduced one of the most stringent lockdowns in Africa, banning cars and public gatherings, shutting down shopping centres, places of worship, schools and entertainment centres, and putting in place a night-time curfew.” (See https://www.telegraph.co.uk/global-health/science-and-disease/ugandas-tough-approach-covid-19-hurting-citizens/ ). There were roadblocks manned by Military Police, the Army and Local defense personnel to ensure compliance. The measures were so strict to the extent of shooting some noncompliant citizens. Some of those acts are reflected in the BBC news article titled “Uganda - where security forces may be more deadly than coronavirus” (see https://www.bbc.com/news/world-africa-53450850 )
The approach of Rawson et al. 2020 in which the population was split into a quarantine and a non-quarantined group differing by the rate of virus transmission and the groups being connected by the modeled release strategies from lockdown seems to be one of the most suitable approaches for the countries in which lockdown restrictions were less strict.
We have added a justification for our choice of lockdown modeling in the discussion on page 18 in lines 380-386 as “On lockdown modeling, we assumed that a percentage of the susceptible population was isolated by the lockdown restrictions and hence impossible to become infected. This assumption is justified by the strictness in enforcing these measures in Uganda. However, for relatively lax implementation of the measures, alternative approaches such as that involving splitting the population into the quarantine and non-quarantine groups as done by [Rawson et al. 2020] could be more suitable.”

This is all excellent contextual information that could be included in a brief paragraph in the introduction to help stress the importance of this work.

When the model is introduced (from L102), it is very difficult to interpret the model initially, needing to jump between table 1, figure 1, equations (1)-(5), and brief paragraphs. I would instead urge presenting each equation, one-by-one, and explaining specifically each term (and parameter) in each equation. This may seem excessive, but makes interpreting a model considerably easier.

The flow of content in this section has been improved up by numbering and then describing each equation fully in the text right below where all the equations appear. The content is capture on page 9 in lines 179-200 as “In Equation (3.1), the term (1-(A+E))ΛN represents the number of arriving susceptible individuals per day, ΒBS(QI_A+I_S+GH)/N represents the number of susceptible individuals that become latently infected per day, ΤR represents the number of previously immune individuals that lose their disease-induced immunity to become susceptible per day and ΛS represents the number of departing susceptible individuals per day. In Equation (3.2), the term EΛN represents the number of arriving latently infected individuals per day while ΡE represents the number of latently infected individuals that progress to become infectious per day. In Equation (3.3), the term AΛN represents the number of arriving asymptomatically infectious individuals per day, RΡE represents the number of latently infected individuals that become asymptomatically infectious per day and Ω_A I_A represents the number of asymptomatically infectious individuals that are hospitalized per day. In Equation (3.4), the term (1-(C+R))ΡE represents the number of latently infected individuals that become symptomatically infectious per day, Σ_S I_S represents the number of symptomatically infectious individuals that succumb to the disease before being hospitalized per day and Ω_S I_S represents the number of symptomatically infectious individuals that are hospitalized per day. In Equation (3.5), the term CΡE represents the number of latently infected individuals that were traced and isolated prior to being hospitalized upon testing positive per day, Ω_A I_A represents the number of asymptomatically infectious individuals that are hospitalized per day, Ω_S I_S represents the number of symptomatically infectious individuals that are hospitalized per day, Σ_H H represents the number of hospitalized individuals that succumb to the disease per day while ΑH represents the number of hospitalized individuals that recover from the disease per day. Lastly, in Equation (3.6), the term ΛR represents the number of departing recovered individuals per day and the other two terms are described above.”

The model is now far easier to read and interpret to new readers, many thanks for these changes.

…

Minor Comments:
L51/L52 – “measures reduced the susceptible population to about 10%”, this is important, is there a citation for this?
This was mentioned in press briefings by the Ministry of Health and other non-documented outlets. However, it can be approximated based on the work demographic data of Uganda (Source: Uganda Bureau of Statistics) where 80% of Ugandan’s workforce is employed in the non-formal sector and hence were less likely to have been categorized as “Essential workers” as per the definition used during lockdown. Only a few market vendors (who were not even allowed to return home to their families) as well as essential, health service providers and essential cargo transporters were allowed to operate. Also of the 43 million Ugandans, 15 million are school going and hence congregate in schools yet all schools were closed during lockdown.

Can you cite this particular press-briefing? Or mention these specific points somewhere in the manuscript to help explain this point.

L129 – Does this mean that asymptomatic individuals are hospitalised? I assume this is due to the test-and-trace, this needs to be changed.
Yes. Everyone that tested positive- symptomatic or not- had to be hospitalized to prevent them from spreading the disease in the community. This went on until recently (as recent as early November 2020) when the hospitals got overwhelmed.

Again, this makes a lot more sense now that I understand the Uganda-specific protocol! Thank you for explaining it.

SUMMARY

The revisions made address almost all of my points very well. There is only one point that needs addressing before I can finally recommend for publication, and that is regarding the model parameter values again. This work will be extremely useful in the coming years I believe for investigating the impact of different mitigation strategies, and as such it is important to make sure that these parameter values are well cited and explained for future readers to understand the measures unique to Uganda.

Essentially I would just want to see some more information for those that cite reference [4]. Naturally, some of these parameters will be deeply uncertain, and so “back-of-the-envelope” estimates will need to be used. There is nothing wrong with this, however I would want to see exactly how these values were reached. As this could involve a lot of text, I would perhaps recommend moving these calculations to a separate appendix or supplementary info page.

Also, where specific press-releases or ministerial briefings are used, these specific briefings should be cited, such as the ones that the authors linked to me in the above comment regarding influx from other countries / lockdown amounts. In short, a lot of the justification given to me in responses by the authors should be included for all readers!

Really the main sticking point here is still the mortality rate. These estimates were based on old data that is clearly now not an accurate representation of the true mortality rate to my eyes. Indeed, based on today’s case data the total mortality rate is 0.007 in Uganda for confirmed cases. The current parameter value used is likely to really affect the results shown, which is important given some of the findings regarding hospital capacity. I think these really need to be re-run with an improved mortality rate, unless my understanding is incorrect?

Once this is addressed I will be happy to recommend for publication.

A minor point, I think it would be extremely helpful for reviewers if a paragraph was included in the Introduction that outlines the specific, extreme, protocols used in Uganda. This motivates the work extremely well, showing why bespoke modelling frameworks are needed.

Finally,

L295 – “Even more worrying is the fact that the endemic level would higher at bigger levels of susceptibility” – I think there’s a typo here, I’m a bit confused about what is trying to be said with this sentence?
